# Recent Advances in Phase-Engineered Photocatalysts: Classification and Diversified Applications

**DOI:** 10.3390/ma16113980

**Published:** 2023-05-26

**Authors:** Jianjian Yi, Guoxiang Zhang, Yunzhe Wang, Wanyue Qian, Xiaozhi Wang

**Affiliations:** 1 College of Environmental Science and Engineering, Yangzhou University, Yangzhou 225127, China211604220@stu.yzu.edu.cn (W.Q.); 2 College of Chemistry and Chemical Engineering, Yangzhou University, Yangzhou 225127, China

**Keywords:** phase engineering, photocatalysis, water splitting, CO_2_ reduction, pollutant degradation

## Abstract

Phase engineering is an emerging strategy for tuning the electronic states and catalytic functions of nanomaterials. Great interest has recently been captured by phase-engineered photocatalysts, including the unconventional phase, amorphous phase, and heterophase. Phase engineering of photocatalytic materials (including semiconductors and cocatalysts) can effectively affect the light absorption range, charge separation efficiency, or surface redox reactivity, resulting in different catalytic behavior. The applications for phase-engineered photocatalysts are widely reported, for example, hydrogen evolution, oxygen evolution, CO_2_ reduction, and organic pollutant removal. This review will firstly provide a critical insight into the classification of phase engineering for photocatalysis. Then, the state-of-the-art development of phase engineering toward photocatalytic reactions will be presented, focusing on the synthesis and characterization methodologies for unique phase structure and the correlation between phase structure and photocatalytic performance. Finally, personal understanding of the current opportunities and challenges of phase engineering for photocatalysis will also be provided.

## 1. Introduction

The crystal phase is an emerging structural parameter of solid materials which holds the key to affecting the functionalities and properties of solid catalytic materials [1,2,3]. With phase transition, different atom arrangements over bulk and surface will lead to the change of physical and chemical properties. As a result, properties such as optical adsorption range, electrochemical conductivity, and molecular adsorption ability can be adjusted, which allows the modulation of function-oriented behaviors for various catalytic applications [4,5,6]. Phase engineering can therefore be defined as a strategy that constructs a specific phase for typical investigative purposes. Many catalytic materials exist in more than one crystal phase, thus providing the possibility of optimizing the performance or broadening the scope of applications. After decades of rapid progress in this research area, great achievements have been made in the phase engineering of nanomaterials, including synthetic methods to realize controllable synthesis of desired phase-engineered materials, fine characterization techniques to clearly analyze the phase structures, and development in various applications [7,8,9,10].

Photocatalysis is considered as an emerging concept of catalysis that can realize the conversion of solar energy to chemical energy [11,12,13,14]. As a green sustainable catalytic technology, the main working mechanism of photocatalysis is exciting semiconductors to generate electron-hole pairs, which can participate in different redox reactions, for example, water splitting [15], CO_2_ reduction [16], and organic pollutant degradation [17]. Nevertheless, the development of photocatalysis up to practical applications still confronts a big challenge, since the performance of the traditional photocatalytic systems is far from satisfactory. Generally, the total photocatalytic process can be divided into six steps, (i) photon absorption, (ii) exciton separation, (iii) carrier diffusion, (iv) carrier transport, (v) catalytic conversion (redox), and (vi) mass transfer of reactants. Additionally, the total conversion efficiency in photocatalytic processes could be generally determined by the efficiency of three main steps, i.e., light absorption efficiency, charge separation efficiency, and surface redox reactivity [18]. The phase engineering of photocatalytic nanomaterials has recently captured great interest in photocatalysis since there are many unique superiorities of phase engineering for tuning photocatalytic behavior regarding the three main steps during photocatalysis. Firstly, phase engineering of semiconducting photocatalysts can provide possibility to adjust the electronic band structures for tunable light absorption ranges. Secondly, phase engineering can change the atomic arrangement in bulk and surface of materials, resulting in the optimization of built-in electric fields. As a result, the charge separation behavior can further be affected. Thirdly, the surface redox reactivity strongly depends on the adsorption/activation ability of reactant molecules, which can be finely tuned by phase-engineering-induced surface atomic reconfiguration [19]. In recent years, various investigations have resulted in improved photocatalysis by rational phase engineering, not limited to phase engineering for pristine semiconductors [20,21] but also for cocatalysts [22,23,24]. Moreover, phase engineering of nanomaterials is not just limited to phase transition from one phase to another. The formations of the amorphous phase [25] and heterophase (e.g., phase junction) [26,27] have also been widely studied.

In this review, we focus on presenting recent advances in phase engineering for photocatalytic reactions. The classification of phase engineering toward photocatalysis is firstly clarified. Then, an overview of state-of-the-art developments in photocatalytic applications based on phase engineering is presented, including but not limited to photocatalytic water splitting, CO_2_ reduction, and pollutant removal. As a focus, the synthesis and characterization methods for desired phase structures and the correlation between phase structure and photocatalytic performance are emphasized. Finally, the current challenges and further opportunities of phase engineering for photocatalysis are envisioned. We try to summarize phase engineering for photocatalytic materials to tackle limited catalytic efficiency and highlight the importance of phase engineering for photocatalysis. The description of representative samples in each catalytic reaction would be sufficient for a general review for researchers who are not familiar with phase-engineered photocatalysts.

## 2. Classification of Phase-Engineered Nanostructures

**Figure 1:**
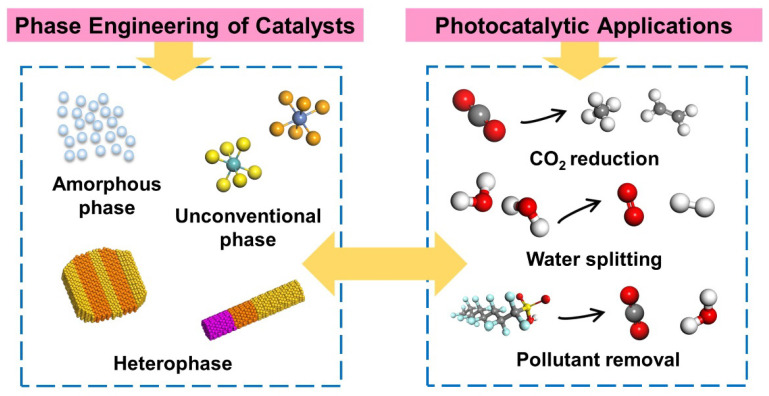
Schematic illustration of different types of phase engineering and the applications in photocatalysis.

Considering that the emerging phase engineering strategies are complicated and diversified, the classification of phase engineering is important for the discussion about phase engineering for photocatalytic applications. As illustrated in Figure 1, we classify the phase-engineered nanostructures into the following three types: the unconventional phase, amorphous phase, as well as the heterophase.

The definition of the unconventional phase is a relative concept to the conventional phase. In general, nanomaterials exist in the form of thermodynamically stable phases in bulky components, which can be defined as the conventional phase. However, nanomaterials with different phases can be obtained by adjusting reaction kinetics and surface energy under certain experimental conditions. The obtained phases with different atom arrangements compared to the conventional phases can be denoted as the unconventional phase. Taking metal as an example, Au is usually crystallized in the conventional face-centered cubic (fcc) phase, but Zhang et al. demonstrated that Au can also be crystallized into 2H and 4H phases by controlling the synthetic parameters [28,29]. In this case, the fcc phase is the conventional phase for Au, while the 2H and 4H phases are unconventional phases. Until now, unconventional phases have been found in a variety of nanomaterials, including metals, metal oxides, and transition metal chalcogenides. The obtained nanomaterials with unconventional phases show unique and enhanced performance effects in many applications.

The amorphous phase is a relative concept to the crystalline phase, with unique structural characteristics. The key feature of the amorphous phase is the short-range order but long-range disorder atomic structure. In contrast, the crystalline phase generally is in the form of short-range orders and long-range orders [30,31]. Due to the disordered arrangement of atoms and high entropy resulting from unsaturated bonds, amorphous materials are usually metastable and readily change to crystalline states under external heat or pressure. The synthesis of amorphous phase nanomaterials can be realized through direct preparation by finely controlling the crystallization process or indirect preparation by breaking the long-range order structure of the crystalline phase [32].

The heterophase structure is different from the mentioned unconventional phase and amorphous phase. It refers to the multi-phase structure composed of two or more crystalline phases of the same material.

The most classical example for the heterophase structure is the commercial TiO_2_ materials, namely P25, which is composed of mixed anatase and rutile phases [33]. Compared to the pristine anatase phase and rutile phase, P25 can exhibit improved photocatalytic activity in many reactions due to the improved charge separation efficiency with the formation of a space-charge layer. In addition to the phase I/phase II heterophase structure, crystalline/amorphous structures can also be defined as heterophase structures [34,35].

With the rational design of material structural engineering based on constructing different phase structures, the electronic structure and catalytic functions can be finely tuned for improved photocatalysis. Different components in a typical photocatalytic material, including light-harvesting semiconductors and surface active cocatalysts, can be tuned based on phase engineering to meet different requirements in different reactions. With the development of phase engineering, phase-dependent properties and photocatalytic applications (e.g., water splitting, CO_2_ reduction and pollutant removal) have been witnessed. Detailed discussion in terms of recent advances in phase engineering for photocatalysis will be provided in the following section.

## 3. Phase Engineering for Photocatalytic Applications

Phase-engineered nanomaterials have endowed them with unique electronic structures and catalytic properties for various applications such as hydrogen evolution, oxygen evolution, CO_2_ reduction, and pollutant removal. Phase engineering can enhance the catalytic performance of typical reactions by broadening the light absorption or steering charge transfer kinetic or by maneuvering the surface redox reaction. In addition, photocatalysts or cocatalysts with different phase structures can also affect the reaction selectivity and stability. In this section, an overview of some recent advances in phase engineering for photocatalytic reactions will be provided.

### 3.1. Hydrogen Evolution

Developing clean and renewable fuels with high energy density is the common pursuit of the academic community. Hydrogen energy is one of the candidates to meet the requirements mentioned above. Photocatalytic water splitting to produce hydrogen is the “holy grail” in solar energy conversion, which is still restricted by the activity, stability, and economic cost [13,36,37]. It has been widely found that phase engineering can significantly boost the photocatalytic hydrogen evolution performance.

**Figure 2:**
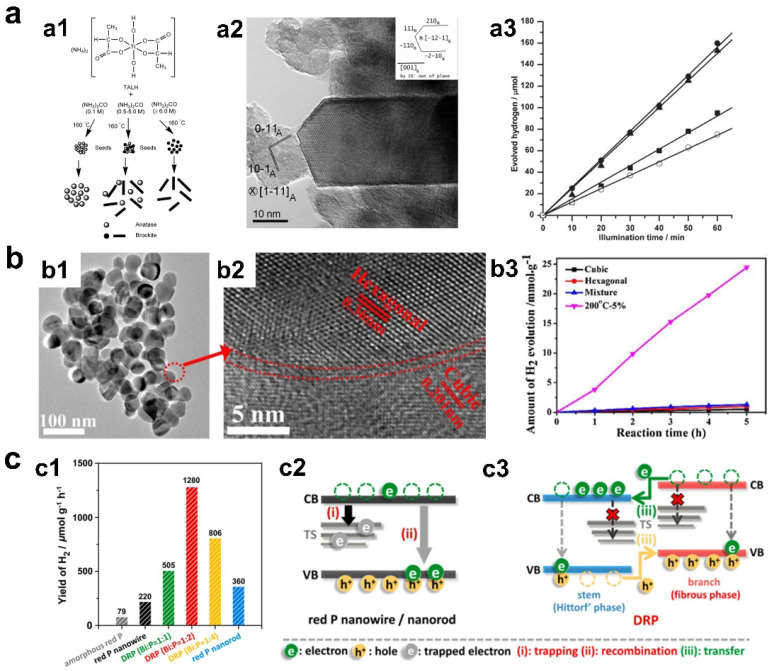
(**a1**) Proposed routes for the formation of anatase and brookite phase TiO_2_. (**a2**) HRTEM image of TiO_2_ with mixed anatase and brookite phase. (**a3**) Photocatalytic hydrogen evolution activity of TiO_2_ with different phase structures using Pt as cocatalyst; ■, ●, ▲, and ○ indicate anatase phase, anatase/brookite mixed phase, brookite phase, and P25, respectively (copyright from Ref. [38], 2010, American Chemical Society). (**b1**,**b2**) TEM and HRTEM images of CdS with mixed hexagonal and cubic phases. (**b3**) Photocatalytic hydrogen evolution activity of the catalysts (copyright from Ref. [40], 2018, Elesvier). (**c1**) Hydrogen product yield of red P samples with different phase structures under visible light irradiation. (**c2**,**c3**) Schematic representation of proposed charge dynamics in (**c2**) red P nanowire/nanorod and (**c3**) heterophase red P with fibrous and Hittorf’s phases (copyright from Ref. [41], 2022, Elesvier).

Recently, phase-engineered semiconducting photocatalysts have been widely designed to modulate their photocatalytic hydrogen evolution performance. Some important electronic structures such as light absorption edges and strength of built-in electric fields can be tuned by phase engineering. Taking the most classic photocatalyst, TiO_2_, as an example, it was reported that the photocatalytic hydrogen evolution performance of brookite phase TiO_2_ was significantly higher than that of anatase phase TiO_2_ (Figure 2a) [38]. The author explained that the conduction band (CB) edge of brookite phase TiO_2_ was more negative than that of anatase TiO_2_ supported by experimental characterizations. Electrons excited at the CB of brookite phase TiO_2_ with higher reduction ability can more effectively reduce H^+^ to H_2_, leading to higher hydrogen evolution activity. Similar cases can also be found in sulfide photocatalysts such as ZnS. Feng et al. reported that the phase structure of ZnS can be regulated by ambient S annealing [39]. The photocatalytic hydrogen evolution measurement showed that wurtzite phase ZnS showed better hydrogen evolution activity than sphalerite phase ZnS. The reason for the phase dependence was owing to the strengthened inter-polar electric field of wurtzite phase ZnS, which could promote the electron-hole separation.

Apart from the formation of a typical phase, the construction of hybrid photocatalysts with heterophase (e.g., phase junction) structure is considered as another effective phase engineering strategy for improved hydrogen evolution catalysis. For instance, CdS, a photocatalyst with desirable bandgap and availability limited by photo-corrosion and inefficiency, was reported to have photocatalytic hydrogen evolution performance that can be optimized by constructing phase junctions [20,40]. Experimental results revealed that the phase junction composed of cubic and hexagonal phase CdS (denoted as c-CdS/h-CdS) showed a high hydrogen evolution rate (4.9 mmol h^−1^ g^−1^) and external quantum efficiency (EQE) of 41.5% at 420 nm (Figure 2b) [40]. The hydrogen evolution rate was 60 times higher than those of c-CdS and h-CdS. Notably, photo-corrosion can also be inhibited over the phase junction. The origin of improved activity and stability was owing to the greatly enhanced charge separation by the regulation of bonding region between cubic and hexagonal phases. Recently, Yu et al. demonstrated an efficient heterophase red P photocatalyst with a hydrogen evolution rate over 1280 μmol h^−1^ g^−1^ (Figure 2c) [41]. The formation of red P heterophases consisting of fibrous and Hittorf’s phases can be realized by Bi-mediated catalytic synthetic method. From the fact that each phase red P possessed different band alignments, the intimate heterophase junction afforded an effective built-in driving force for efficient charge transport, thus achieving high catalytic performance. Similar case studies were also demonstrated over TiO_2_ [34], phosphorus [42], Cd_1−x_Zn_x_S [43], ln_2_O_3_ [44], and ZnIn_2_S_4_ [45], highlighting the advance of heterophase structures.

**Figure 3:**
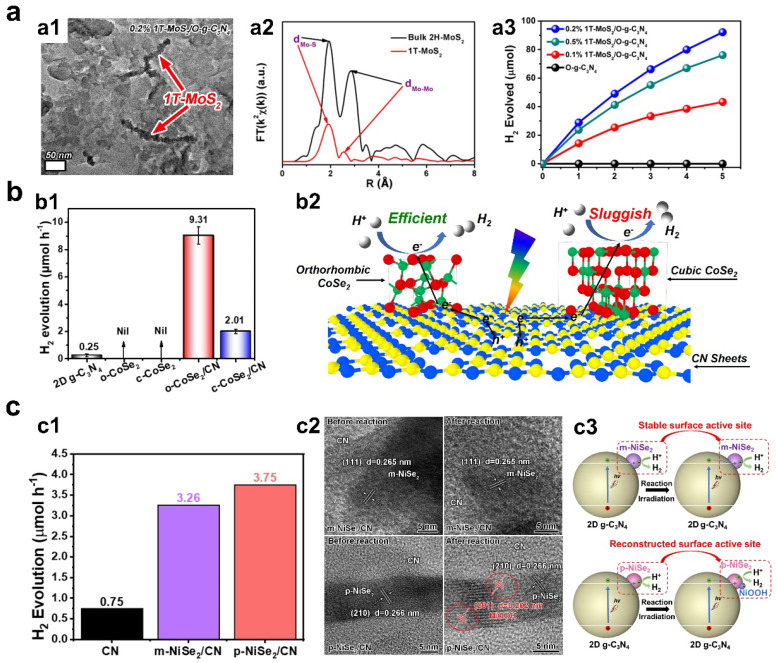
(**a1**) TEM image of 1T-MoS_2_/O-g-C_3_N_4_. (**a2**) Fitted EXAFS results of prepared 1T-MoS_2_ sample and commercial 2H-MoS_2_. (**a3**) Time-dependent hydrogen evolution rate of the samples (copyright from Ref. [48], 2018, Elesvier). (**b1**) A comparison of light-driven hydrogen evolution rate of CoSe_2_-based catalysts. (**b2**) Charge behavior of o-CoSe_2_/CN and c-CoSe_2_/CN (copyright from Ref. [52], 2020, Elesvier). (**c1**) A comparison of light-driven hydrogen evolution rate of NiSe_2_-based catalysts. (**c2**) HRTEM images of NiSe_2_/CN samples before and after photocatalysis. (**c3**) Mechanism illustration of phase-dependent stability of NiSe_2_ (copyright from Ref. [54], 2023, Elesvier).

Transitional metal dichalcogenides (TMDs) represented by MoS_2_ show potential to improve photocatalytic hydrogen evolution performance as cocatalysts on semiconductor surfaces. TMD nanomaterials show various phases owing to the different electronic structures of transition metal atoms with different d orbital filling states [22,46,47]. For example, 2H-MoS_2_ and 2H-WS_2_ are semiconducting with band gaps, whereas 1T-MoS_2_ and 1T-WS_2_ are metallic with good conductivity for hydrogen evolution reactions. Our group found that 1T-MoS_2_/O-g-C_3_N_4_ demonstrated a greatly higher photocatalytic hydrogen evolution performance compared to 2H-MoS_2_/O-g-C_3_N_4_ (Figure 3a) [48]. The optimal 1T-MoS_2_/O-g-C_3_N_4_ sample showed hydrogen evolution rate over 1800 μmol/g/h with external quantum efficiency of 7.11% at 420 nm. The origin for the high catalytic activity of 1T-MoS_2_ can be ascribed to the metal-like conductivity and the active edge and basal sites for hydrogen evolution. By contrast, low conductivity and limited active sites at edge sites leads to poor performance. Recent studies also reported that 1T-MoS_2_, 1T-WS_2_, and 1T-MoSe_2_ can improve the catalytic activity of hosting semiconductors not limited to g-C_3_N_4_ but also other semiconductors such as TiO_2_ and CdS [23,24,49,50,51].

In addition to MoS_2_ with layered structure, TMD materials with non-layered structures such as CoSe_2_ and NiSe_2_ also show phase-dependent catalytic hydrogen evolution performance. Our group demonstrated the phase-dependent photocatalytic hydrogen evolution catalysis of CoSe_2_. In a practical photocatalytic process, it was observed that CoSe_2_ with orthorhombic phase (o-CoSe_2_) can better improve the hydrogen evolution rate of g-C_3_N_4_ semiconductors than CoSe_2_ with cubic phase (c-CoSe_2_) (Figure 3b) [52]. It was revealed by density functional theory (DFT) calculations that the Co site on o-CoSe_2_ surface showed more appropriate hydrogen adsorption Gibbs free energy (∆G_H*_ = 0.27 eV) than the c-CoSe_2_ surface, resulting in improved catalysis. Despite c-CoSe_2_ possessing better conductivity than o-CoSe_2_, the charge separation efficiency may not be the rate-determining step in this case. Interestingly, unlike the higher catalytic performance of o-CoSe_2_/g-C_3_N_4_ compared to c-CoSe_2_/g-C_3_N_4_, we recently found that the hydrogen evolution of o-CoSe_2_/TiO_2_ (2.601 µmol/h) was lower than that of c-CoSe_2_/TiO_2_ (12.001 µmol/h) [53]. We propose the reason for this phenomenon would be that the interfacial charge transfer between TiO_2_ and c-CoSe_2_ is the dominating factor but not the surface redox reactivity. Impressively, phase engineering of TMD-based cocatalysts show not only phase-dependent activity but also stability. Very recently, we reported that the phase structure of NiSe_2_ played important role in determining the photocatalytic hydrogen evolution stability instead of stability (Figure 3c) [54]. Upon light irradiation on m-NiSe_2_/CN and p-NiSe_2_/CN in TEOA/H_2_O, comparable photocatalytic hydrogen evolution rates of 3.26 μmol h^−1^ and 3.75 μmol h^−1^ can be observed. Importantly, we found that NiSe_2_ exhibited phase-dependent stability, i.e., m-NiSe_2_ can evolve H_2_ steadily, but p-NiSe_2_ showed a ~57.1% rate decrease after 25 h of reaction. After fine characterization, we proposed the origin of phase-dependent stability. The chemical structure of m-NiSe_2_ can be well preserved in a catalytic process, but partial p-NiSe_2_ tends to be converted to NiOOH, resulting in different catalytic stability.

### 3.2. Oxygen Evolution

Photocatalytic water splitting to produce hydrogen or CO_2_ reduction to obtain high value-added chemical products are ideal pathways to achieve solar energy storage and conversion. In the overall reactions, water oxidation is the most important half reaction of these two energy photocatalytic reactions [55,56,57]. In water splitting and CO_2_ reduction reactions, water oxidation provides protons and electrons, which is the premise of the reduction reaction. It should be noticed that the water oxidation reaction is a four-electron reaction, and the overpotential of this reaction is very high. It is thus considered as the rate-determining step of the overall reactions, which holds the key to the proceeding of hydrogen evolution from water or CO_2_ reduction [58,59]. Phase engineering of nanomaterials could improve photocatalytic oxygen evolution performance by enhancing charge separation, or decreasing the reaction energy barrier.

**Figure 4:**
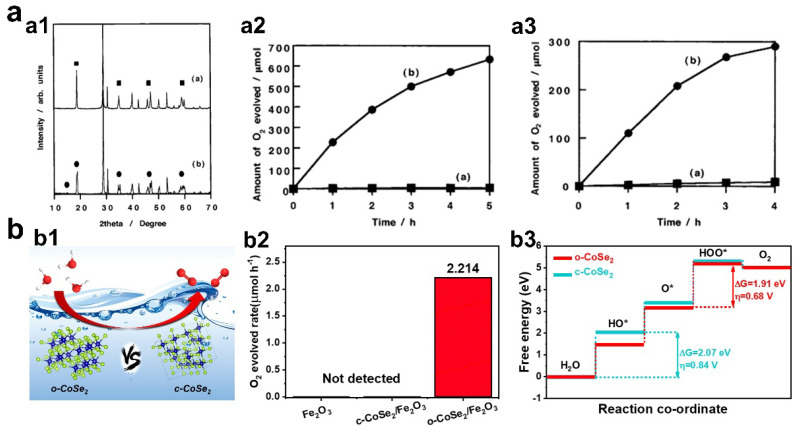
(**a1**) XRD patterns of BiVO_4_ with different phases, ■ and ● indicate tetragonal and monoclinic phase respectively. (**a2**,**a3**) Photocatalytic oxygen evolution performance of BiVO_4_ samples under visible light irradiation (λ > 420 nm) and under ultraviolet light (300 nm < λ < 380 nm). Inset: (**a**)—tetragonal phase, (**b**)—monoclinic phase (copyright from Ref. [60], 2001, American Chemical Society). (**b1**) Schematic of oxygen evolution process over CoSe_2_ with different phase structures. (**b2**) Photocatalytic oxygen evolution performance of the catalysts. (**b3**) Diagram of the calculated free energy change in each reaction step over o-CoSe_2_ and c-CoSe_2_ (copyright from Ref. [62], 2022, Elesvier).

Phase-dependent photocatalytic performance was reported over a classical oxygen evolution semiconducting photocatalyst, i.e., BiVO_4_. Kudo et al. synthesized tetragonal phase and monoclinic phase BiVO_4_ successfully, characterized the phase and optical structures, and measured the photocatalytic O_2_ evolution performance in AgNO_3_ solution (Figure 4a) [60]. Given the similar light harvesting capacity of tetragonal BiVO_4_ and monoclinic BiVO_4_, it was interesting that negligible O_2_ gas product can be detected over tetragonal BiVO_4_, but monoclinic BiVO_4_ exhibited high O_2_ evolution activity (over 120 μmol/h under visible light and over 70 μmol/h under UV light). Mechanism analysis revealed that distortion of a Bi-O polyhedron by a 6s^2^ lone pair of Bi^3+^ in monoclinic BiVO_4_ was beneficial for the surface conversion from H_2_O to O_2_, leading to high photocatalytic activity. In another research work conducted by Amal et al., the authors investigated the amorphous and crystalline evolution of BiVO_4_ during the synthesis by flame spray pyrolysis. In terms of the photocatalytic test, the first finding was that amorphous BiVO_4_ cannot produce O_2_ by photocatalysis. For crystalline BiVO_4_, the photocatalytic oxygen evolution rate increased with the increased content of monoclinic phase in BiVO_4_, highlighting the important role of monoclinic phase [61].

Except for phase engineering of semiconductors, phase-dependent oxygen evolution photocatalysis was also found in surface active cocatalysts. For example, our research group demonstrated that the phase structure of CoSe_2_ cocatalysts plays an important role in determining the oxygen evolution performance of Fe_2_O_3_ semiconductors (Figure 4b) [62]. Experimental results found that orthorhombic phase CoSe_2_ (o-CoSe_2_) showed better potential than cubic phase CoSe_2_ (c-CoSe_2_) in enhancing photocatalytic oxygen evolution performance of Fe_2_O_3_. o-CoSe_2_/Fe_2_O_3_ can realize the qualitative changes of oxygen evolution rate from ‘‘0” to ‘‘1” under visible light irradiation, using AgNO_3_ as sacrificial agent and La_2_O_3_ as pH balance agent. However, c-CoSe_2_/Fe_2_O_3_ cannot work in photocatalytic oxygen evolution processes under the same conditions. Combined with photoelectrochemical characterization and theoretical simulations, we proposed that the key factor for the superior activity of o-CoSe_2_ was the decreased activation barrier of H_2_O on its surface.

### 3.3. CO_2_ Reduction

Converting CO_2_ into valuable chemical feedstocks or liquid fuels under mild conditions is of great significance for reducing the greenhouse effect and achieving carbon neutrality [63,64,65]. Among them, photocatalysis is considered a green technology that converts CO_2_ from solar energy [66,67]. The key steps of photocatalytic CO_2_ reduction include the generation of electron-hole (e-h) pairs, electron transfer to catalytic active sites, and catalytic CO_2_ reduction. In order to meet these requirements, the rational structural design of catalysts is required [68]. Due to the ability to optimize the multiple electronic structures of catalysts, especially the regulation of CO_2_ molecular adsorption activation behavior, phase engineering in the field of CO_2_ photoreduction has also attracted extensive attention [69].

**Figure 5:**
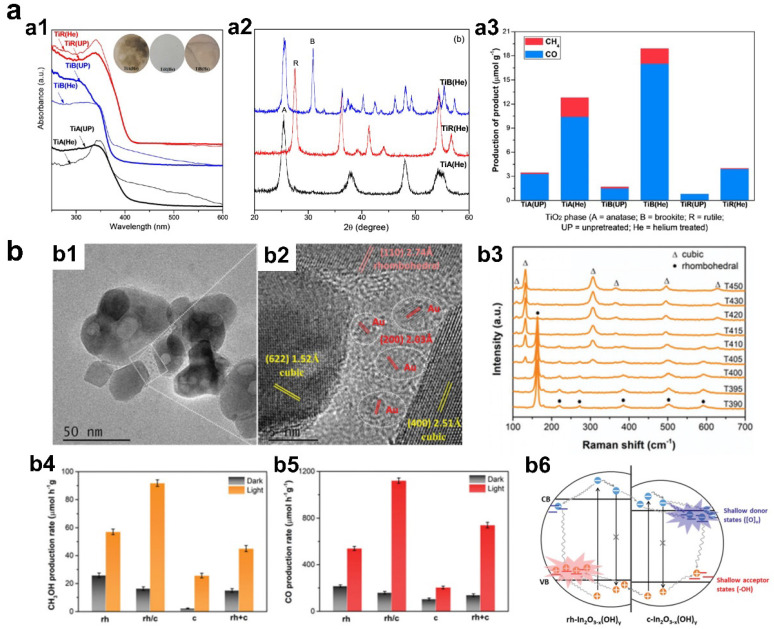
(**a1**) UV-visible diffuse reflectance spectra of unpretreated and He-pretreated TiO_2_ (anatase, rutile, and brookite). (**a2**) XRD patterns of He-pretreated TiO_2_ (anatase, rutile, and brookite). (**a3**) Photocatalytic CO_2_ reduction performance of TiO_2_ with different phases (copyright from ref. [70], 2012, American Chemical Society). (**b1**,**b2**) TEM and HRTEM images of ln_2_O_3−X_(OH)_y_ composed of rhombohedral and cubic phase. (**b3**) XRD patterns illustrating the phase transition from rhombohedral to cubic phase with increased temperature. (**b4**,**b5**) CH_3_OH and CO evolution rates over the catalysts with different phase structures. (**b6**) Schematic illustration of charge carrier separation and recombination pathways in polymorphic heterostructures of rh/c-In_2_O_3−x_(OH)_y_ (copyright from ref. [71], 2020, Royal Society of Chemistry).

Phase engineering of pristine semiconductor has shown potential in tuning the photocatalytic CO_2_ reduction performance. Li et al. reported the phase-controlled synthesis of three types of TiO_2_ (anatase, rutile, and brookite), and the evaluation of photocatalytic CO_2_ reduction performance with water vapor (Figure 5a) [70]. Experimental results found that the production of CO and CH_4_ using anatase and brookite phase TiO_2_ were enhanced by 10-fold in contrast to rutile phase TiO_2_. The new finding in this study is that brookite phase TiO_2,_ with less attention among the three TiO_2_ polymorphs, exhibited the highest CO and CH_4_ production rates. Detailed mechanism studies revealed that the superior activity of brookite phase TiO_2_ may be owing to the existence of surface oxygen vacancies, faster reaction rate of CO_2_^−^ with surface adsorbed H_2_O or OH groups, and a new reaction pathway involving an HCOOH intermediate.

In addition to the complete phase transition in semiconductor photocatalyst, the construction of heterophase photocatalyst recently attracts intensive attention in CO_2_ reduction. Yan et al. reported a heterophase photocatalyst based on ln_2_O_3−X_(OH)_y_ to optimize the CO_2_ reduction performance (Figure 5b) [71]. The continuous transition from cubic to rhombohedral can be realized by temperature variety so that the heterophase structure can be obtained by partial phase transition at an appropriate temperature. The charge separation efficiency can be greatly improved due to the formation of the cubic/rhombohedral interface. As a result, the optimized cubic/rhombohedral ln_2_O_3−X_(OH)_y_ exhibited improved CO_2_ reduction activity compared to that of the pure cubic or rhombohedral phase, with a CH_3_OH evolution rate of 92 μmol/g/h and CO evolution rate of 1120 μmol/g/h. This strategy can also be extended to other material systems with similar working mechanisms for CO_2_ reduction, for example, CulnS_2_ [72] and CdS [73].

**Figure 6:**
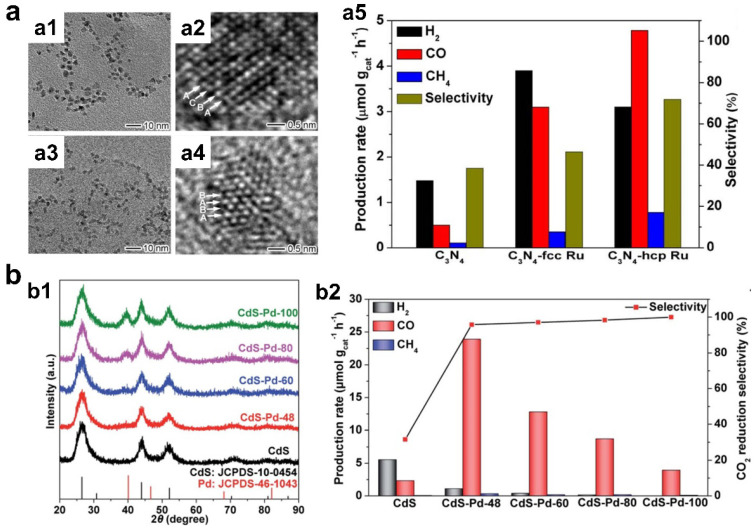
(**a1**,**a2**) TEM and HRTEM images of C_3_N_4_-fcc Ru. (**a3**,**a4**) TEM and HRTEM images of C_3_N_4_-hcp Ru. (**a5**) Photocatalytic CO_2_ reduction activity and selectivity (copyright from ref. [74], 2018, Elesvier). (**b1**) XRD patterns of CdS-Pd samples. (**b2**) Photocatalytic CO_2_ reduction activity and selectivity (copyright from ref. [75], 2020, Royal Society of Chemistry).

In photocatalytic hybrid structures, cocatalysts are widely used to improving the activity and selectivity of CO_2_ reduction by promoting the electron-hole separation and providing highly active catalytic sites for CO_2_ catalytic conversion. Phase structure engineered metal cocatalysts are recently known as efficient cocatalysts for CO_2_ reduction. For instance, Bai et al. reported that the Ru cocatalyst with hexagonal close-packed (hcp) phase (hcp Ru) can more effectively boost CO_2_ reduction efficiency of C_3_N_4_ semiconductor in contrast to face-centered cubic (fcc) phase Ru (fcc Ru) (Figure 6a) [74]. Thanks to phase engineering of Ru, not only CO and CH_4_ evolution rates but also selectivity for carbon-based products can be improved over C_3_N_4_-hcp Ru. To uncover the mechanism for phase-dependent activity and selectivity, the authors explained this phenomenon supported by electrochemical characterizations and theoretical calculations. The improved performance was not owing to the altered interfacial electron transfer from C_3_N_4_ to Ru, but the higher CO_2_ adsorption energy on (10$\overset{-}{1}$1) face of hcp Ru was higher than that on the (111) face of fcc Ru. Except for the design of an unconventional phase, amorphous phase cocatalysts can also work in CO_2_ reduction reaction with optimized performance. In another case, also reported by Bai et al., the effect of crystallinity on photocatalytic CO_2_ reduction performance was systematically studied by using Pd nanosheets as model cocatalysts (Figure 6b) [75]. When Pd nanosheets were assembled with CdS quantum dots (QDs), it was found that Pd with high crystallity and good lattice periodicity was more conducive to electron transfer from CdS to Pd, which more effectively inhibited H_2_ production on the surface of CdS. In contrast, low-crystallinity Pd provided a large number of surface unsaturated atoms and defects as highly active centers for efficient CO_2_-CO/CH_4_ conversion. The formation rates of CO (23.93 μmol/g/h) and CH_4_ (0.35 μmol/g/h) in CdS-Pd-48 with low-crystallinity Pd were 10.3 and 5.9 times of those of the pristine CdS QDs, respectively. This result highlighted that amorphous-phase Pd contributed to efficient CO_2_ adsorption and activation, and crystalline Pd favored charge migration. Taken together, amorphous Pd was a better cocatalyst for CO_2_ reduction in terms of the yield and selectivity of carbon products.

### 3.4. Pollutant Removal

Semiconductor photocatalysis has been widely applied in environmental remediation. Upon light irradiation, photocatalysts can harvest solar energy to generate electron-hole (e-h) pairs for pollutant removal. Various reactive oxygen species, including O_2_^−^, OH, and ^1^O_2_, can be generated by different redox reactions. In addition, the photogenerated holes can also directly oxidize organic pollutants. All the mentioned reactive species generated by photocatalysis can contribute to efficient pollutant removal [18,76,77]. Phase engineering of photocatalysts can improve the pollutant removal performance by several working mechanisms, for example, decreasing the formation energies of oxygen species, improving the light absorption range, promoting charge separation, improving reduction and oxidation potentials of electrons or holes, and so on.

**Figure 7:**
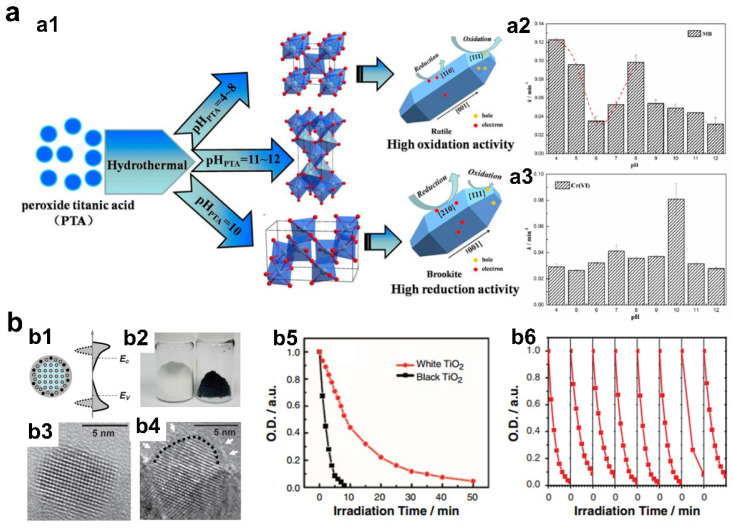
(**a1**) Schematic illustration of synthetic process of TiO_2_ with different phases. (**a2**) Photocatalytic performance of MB degradation. (**a3**) Photocatalytic performance of Cr (VI) reduction (copyright from ref. [78], 2015, American Chemical Society). (**b1**) Schematic illustration of the structure and electronic DOS of a semiconductor in the form of a disorder engineered nanocrystal with dopant incorporation. (**b2**) Digital graphs of unmodified white TiO_2_ and black TiO_2_ with amorphous surface layer. (**b3**,**b4**) HRTEM images of unmodified white TiO_2_ and black TiO_2_ with amorphous surface layer. (**b5**) Photocatalytic MB degradation performance. (**b6**) Stability tests (copyright from ref. [81], 2011, American Association for the Advancement of Science).

Phase-controlled synthesis of TiO_2_ nanorods can be realized by a hydrothermal method using peroxide titanic acid solution with different pH values, forming rutile, anatase, and brookite phase TiO_2_, respectively (Figure 7a) [78]. The phase-dependent photocatalytic activities of the samples were evaluated by reduction of Cr (VI) and degradation of methylene blue (MB). Experimental results indicated that rutile TiO_2_ possessed best photo-degradation performance of MB, while brookite TiO_2_ showed best activity for Cr (VI) photo-reduction. The improved photo-oxidation performance can be explained that rutile TiO_2_ can expose more {111} facets with high surface energy, which can boost the oxidation reaction more easily. For photo-reduction reaction, the more negative CB potential of brookite TiO_2_ contributed to more effective Cr (IV) reduction. This case study highlights the importance of phase design for different environmental photocatalytic reactions.

In many recent studies, it has been proven that amorphization of catalysts is an effective method to tune the physical/chemical properties and thus modulate their photocatalytic degradation performance [79,80]. For example, Mao et al. reported a classical study about increasing light absorption of TiO_2_ by amorphization, forming the so-called black TiO_2_ (Figure 7b) [81]. The researchers demonstrated that the color of white TiO_2_ changed to black for surface amorphization by hydrogenation treatment, leading to light absorption broadening from ultraviolet region to near-infrared region. In a practical photocatalytic degradation of methylene blue (MB) solution, black TiO_2_ with crystalline core and amorphous surface showed significantly improved degradation rate with high stability compared to that of white TiO_2_. The origin for the phase-dependent performance was mainly owing to the change of electronic and optical properties of black TiO_2_, especially the greatly reduced band gap promoted by the formation of midgap states. Similarly, highly efficient photocatalytic degradation can also be demonstrated over other semiconductors though phase transition to amorphous phase by virtue of the high surface energy and outstanding adsorption/desorption properties. Hu et al. reported the synthesis of a series of phase-engineered Sb_2_S_3_ photocatalysts with different degrees of amorphization by adjusting the concentration of hydrochloric acid in hydrothermal processes [82]. The amorphous Sb_2_S_3_ exhibited the best photocatalytic methyl orange degradation activity, which was 13 times higher than that of crystalline Sb_2_S_3_.

**Figure 8:**
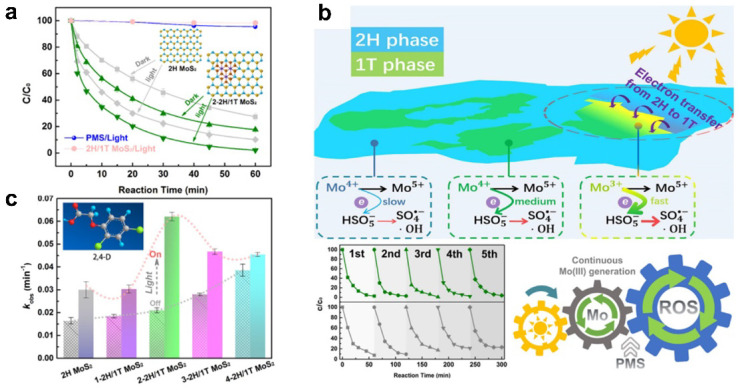
(**a**) Removal efficiency of 2,4-D in different reaction systems. (**b**) Rate constants of 2,4-D degradation using different MoS_2_ samples with (red dashed line) and without (gray dashed line) light irradiation. (**c**) Proposed mechanism for the photoinduced PMS activation on the surface of multiphase MoS_2_ (copyright from ref. [86], 2019, American Chemical Society).

Except for the direct photocatalytic degradation of pollutants, phase engineering in photo-assisted environmental catalysis has recently attracted wide attention [83,84,85]. For instance, the Qu research group reported the construction of heterophase MoS_2_, i.e., 2H/1T MoS_2_, and its application in photo-assisted permonosulfate (PMS) activation for water pollutant degradation (Figure 8) [86]. Although MoS_2_ has been proven as an efficient activator of PMS, it is still restricted by the loss of low-valence Mo during the catalytic process. The authors found that the integration of semiconducting 2H phase MoS_2_ and 1T phase MoS_2_ forming 2H/1T MoS_2_ can favor the catalytic reaction, by introducing photogenerated electrons of 2H MoS_2_ under light irradiation to trigger the formation of low-valence Mo. In this case, the key of this reaction is the regeneration of low valence Mo in 1T MoS_2_. 2H MoS_2_ with semiconductor characteristic in heterophase MoS_2_ can transfer electrons to 1T MoS_2_ under light irradiation, leading to the reduction of high-valence Mo to low-valence Mo (active center in PMS activation). As a result, 2H/1T phase MoS_2_ showed efficient and continuous degradation of organic pollutants in the existence of PMS and light. The discovery in this work highlighted the merits of constructing heterophase structure in heterogeneous photocatalytic degradation reactions.

## 4. Conclusions and Perspectives

In summary, this review summarizes the advances in phase engineering for photocatalytic applications, aiming to outline the route for designing efficient photocatalysts based on phase engineering. Table 1 summarizes some representative nanomaterials with phase engineering and their applications in photocatalysis. Phase-engineered materials can exist in different forms, including the unconventional phase, amorphous phase, and heterophase. Phase engineering can also be performed on both light-harvesting semiconductor and surface active cocatalyst. With optimized electronic structures and physico-chemical properties, the light absorption, charge separation, or surface redox reaction behavior can be tuned, providing the possibility for improved photocatalysis. Phase engineering has gained huge success in the photocatalysis research community, owing to the positive role in various energy and environmental applications.

Although considerable success has been witnessed, there are still some challenges in the research field. Firstly, phase engineering has made surprising progress in enhancing catalyst efficiency, including activity, selectivity, and stability, but record-breaking high-performance photocatalytic efficiency is rarely reported. For example, 1T-MoS_2_ has shown great potential in hydrogen evolution, but the performance is still far from noble metal catalysts such as Pt. Secondly, the phase purity is a general concern in this research field. The incomplete phase transition or unsatisfied synthetic methods are still limiting the phase purity of phase-engineered nanostructures. For instance, 1T-MoS_2_ with pure 1T phase is hard to obtain. Most of the reported studies are 1T/2H mixed phase. Additionally, the phase stability should also be focused. Thirdly, the delicate control over other structural parameters in addition to phase is still challenging. Different morphology, size, or other structural parameters of phase-engineered materials make it difficult to reveal the mechanism. For example, TiO_2_ with different phase structures but also different sizes would make it difficult to determine whether the increase in catalytic activity is due to the crystal phase structure or size.

Despite the presented challenges, there are plenty of opportunities in phase engineering for photocatalytic applications. Firstly, based on the fast development of material science, synthesizing novel nanomaterials such as MOFs and COFs with different phase structures is promising. Secondly, given that phase-engineered photocatalysts are now mainly used in limited applications, exploring new energy and environmental applications such as organic synthesis, H_2_O_2_ production, and N_2_/NO_3_ reduction may broaden the functions. Thirdly, the integration of phase engineering to other structural engineering strategies may make it possible to construct record-breaking high performance photocatalytic systems, and fourth, the development of facile and scale-up synthetic methods is of great significance in future research, with aims to realizing large scale synthesis of phase-engineered materials with high pure purity and stability. Last but not least, from the mechanism point of view, using advanced characterization technologies such as in situ XRD, in-situ Raman spectroscopy, and in situ TEM to uncover the phase evolution mechanism is still under development.

## Figures and Tables

**Table 1:** Summary of some representative phase-engineered photocatalysts and their applications.

Material	Phase	Application	Performance	Ref.
TiO_2_	Brookite	HER	Brookite > Anatase	[38]
CdS	Hexagonal	HER	Hexagonal > Cubic	[40]
Red P	Hittorf/fibrous	HER	Heterophase > Single phase	[41]
MoS_2_	1T	HER	1T > 2H	[48]
CoSe_2_	Orthorhombic	HER	Orthorhombic > Cubic	[52]
NiSe_2_	Marcasite	HER	Marcasite > Pyrite (stability)	[54]
BiVO_4_	Monoclinic	OER	Monoclinic > Tetragonal	[60]
CoSe_2_	Orthorhombic	OER	Orthorhombic > Cubic	[62]
TiO_2_	Brookite	CO_2_ RR	Brookite > Anatase > Rutile	[70]
ln_2_O_3-X_(OH)_y_	Rhombohedra/cubic	CO_2_ RR	Heterophase > Single phase	[71]
Ru	hcp	CO_2_ RR	hcp > fcc	[74]
Pd	Amorphous	CO_2_ RR	Amorphous > Crystalline	[75]
TiO_2_	Amorphous/Crystalline	MB Degradation	Heterophase > Single phase	[81]
MoS_2_	1T/2H	PMS actication	Heterophase > Single phase	[86]

## Data Availability

Raw data are available upon request.
